# Imaging in Colorectal Cancer: Progress and Challenges for the Clinicians

**DOI:** 10.3390/cancers8090081

**Published:** 2016-08-31

**Authors:** Eric Van Cutsem, Henk M. W. Verheul, Patrik Flamen, Philippe Rougier, Regina Beets-Tan, Rob Glynne-Jones, Thomas Seufferlein

**Affiliations:** 1 Department of Gastroenterology/Digestive Oncology, University Hospitals Gasthuisberg Leuven and KU Leuven, 3000 Leuven, Belgium; 2 Division of Medical Oncology, VU University Medical Centre, 1081 HV Amsterdam, The Netherlands; h.verheul@vumc.nl; 3 Nuclear Medicine Imaging and Therapy Department, Institut Jules Bordet, Université Libre de Bruxelles, 1000 Brussels, Belgium; patrick.flamen@bordet.be; 4 Gastroenterology and Digestive Oncology Department, European Hospital, Georges Pompidou, 75015 Paris, France; rougier.philippe2012@gmail.com; 5 Department of Radiology, The Netherlands Cancer Institute, 1066 CX Amsterdam, The Netherlands; beetstan@me.com; 6 Department of Medical Oncology, Mount Vernon Centre for Cancer Treatment, HA6 2RN Middlesex, UK; rob.glynnejones@nhs.net; 7 Clinic of Internal Medicine I, University Hospital Ulm, 89081 Ulm, Germany; Thomas.Seufferlein@uniklinik-ulm.de

**Keywords:** metastatic colorectal cancer, imaging, angiogenesis

## Abstract

The use of imaging in colorectal cancer (CRC) has significantly evolved over the last twenty years, establishing important roles in surveillance, diagnosis, staging, treatment selection and follow up. The range of modalities has broadened with the development of novel tracer and contrast agents, and the fusion of technologies such as positron emission tomography (PET) and computed tomography (CT). Traditionally, the most widely used modality for assessing treatment response in metastasised colon and rectal tumours is CT, combined with use of the RECIST guidelines. However, a growing body of evidence suggests that tumour size does not always adequately correlate with clinical outcomes. Magnetic resonance imaging (MRI) is a more versatile technique and dynamic contrast-enhanced (DCE)-MRI and diffusion-weighted (DW)-MRI may be used to evaluate biological and functional effects of treatment. Integrated fluorodeoxyglucose (FDG)-PET/CT combines metabolic and anatomical imaging to improve sensitivity and specificity of tumour detection, and a number of studies have demonstrated improved diagnostic accuracy of this modality in a variety of tumour types, including CRC. These developments have enabled the progression of treatment strategies in rectal cancer and improved the detection of hepatic metastatic disease, yet are not without their limitations. These include technical, economical and logistical challenges, along with a lack of robust evidence for standardisation and formal guidance. In order to successfully apply these novel imaging techniques and utilise their benefit to provide truly personalised cancer care, advances need to be clinically realised in a routine and robust manner.

## 1. Introduction

Colorectal cancer (CRC) is among the most commonly diagnosed cancers worldwide. In 2012, there were 1,360,000 new cases of CRC [1], 447,000 of which were in Europe [2]. CRC is the second most common cancer in this region, accounting for 13% of cancer diagnoses (excluding non-melanoma skin cancers) and 215,000 deaths [2].

Risk factors for CRC include dietary, hereditary and environmental influences [3], which lead to the gradual accumulation of genetic mutations and epigenetic alterations that drive the development of tumours over decades [4]. More than 80% of CRCs arise from adenomatous polyps but less than 1% of adenomatous polyps smaller than 1 cm ever become malignant [5]. Advances in our ability to detect developing CRC has begun to refine the prognostic information available and define patient groups that are likely to benefit from systemic treatment or targeted therapies [6].

The use of imaging in CRC has significantly evolved over the last twenty years [7], establishing important roles in surveillance, diagnosis, staging, treatment selection and follow up [3,8]. The range of imaging modalities currently available for the detection and assessment of tumours can be broadly grouped into two categories: anatomical and functional. Anatomical imaging techniques remain the mainstay, in particular computed tomographic (CT) imaging for colon tumour staging and magnetic resonance imaging (MRI) for rectal tumour staging. In addition, rapidly-evolving molecular imaging techniques such as fluorodeoxyglucose-positron emission tomography (FDG-PET/CT) and recently-developed functional MRI techniques may provide insights into tumour perfusion, metabolic and molecular phenotypes [9]. Such information is becoming more relevant as we begin to understand the cytological mechanisms of tumour response that mediate clinical benefit, with a significant focus beyond the tumour shrinkage that is classically-associated with chemotherapy response [9].

The range of available imaging modalities has broadened significantly over the last two decades as a result of technological diversification, the development of novel tracer and contrast agents, and the fusion of technologies such as PET and CT [10]. No single modality is unequivocally superior for evaluating CRC, which drives the evolution of imaging technologies [5], but may also be responsible for variation in practice, which has been reported within both countries and institutions [11].

Here we review the current status of imaging in CRC, outlining the strengths and limitations of key modalities in the various settings that guide disease management, in particular the challenges faced when evaluating the response of tumours to novel treatments.

## 2. Imaging Modalities for Treatment Optimisation

### 2.1. Assessment beyond RECIST

Accurate evaluation of treatment response is critical for optimal treatment decisions in CRC [12]. Internationally-recognised criteria for the quantification of tumour response in clinical trials have been developed by the World Health Organization (WHO) and the Response Evaluation Criteria in Solid Tumours (RECIST) group to optimise comparability within and between studies [13,14]. The RECIST definition of tumour response (a 30% decrease in unidimensional measurement) is based on the extent of tumour size reduction as measured by anatomical imaging modalities such as CT or magnetic resonance imaging (MRI; Table 1) [13].

The most widely used modality for assessing treatment response in metastasised colon and rectal tumours is CT [15]. Modern multidetector scanners acquire ultrathin slices to allow visualisation in any plane with exceptional spatial resolution [5]. In addition, dynamic contrast enhanced CT (DCE-CT) is also a validated technique, which can assess angiogenesis non-invasively and has been utilised in phase I/II trials of anti-angiogenic drugs, and monitoring disease control after loco-regional therapy [16,17]. Alterations in DCE-CT parameters may occur before any morphological changes in response to therapy, making perfusion CT a further potential surrogate marker of response. There are practical advantages which include the widespread availability of CT, and protocols can be integrated with short coverage periods and hence patient compliance.

As an anatomical, size-based modality, multidetector CT evaluation has conventionally made use of the RECIST guidelines [15]. These criteria are useful when assessing agents that operate via the mechanism of tumour shrinkage. However, a growing body of evidence suggests that tumour size does not adequately correlate with clinical outcomes when assessing treatments that operate via other mechanisms, including molecular targeted treatments and immunotherapies [9,18].

The success or failure of therapeutic agents is identified by clinical outcomes. Tumour shrinkage correlates well with survival benefit for treatments that operate via a cytolytic, mass-reducing action, but this is not the case for drugs that operate via cytostatic mechanisms [19]. An example of a cytostatic mechanism is the anti-angiogenic activity of bevacizumab: inhibiting the growth of new blood vessels does not immediately lead to a decrease in tumour size [8]. This lack of tumour size reduction, despite the presence of clinical benefit, renders RECIST v1.1 criteria less adequate to assess the efficacy of such treatments [20].

The modification of CT structure in metastatic CRC (mCRC) patients treated with bevacizumab is reported to be a more-relevant reflection of tumour response than a change in tumour size. Morphological changes include a transformation of liver metastases from heterogeneous lesions with thick, irregular borders into bland, homogeneously hypodense masses with sharp interfaces between the tumour and adjacent liver parenchyma [21]. These structural criteria correlate strongly with the percentage of residual tumour cells and also with overall survival, whereas tumour size determined by RECIST v1.1 does not [21]. Several alternative treatment response criteria have now been proposed that take tumour morphology into account as well as tumour size [15].

The activity of immunotherapeutic agents may also be inadequately assessed by conventional response criteria, as progressive disease (assessed by radiographic evaluation) does not necessarily reflect therapeutic failure [14]. Tumours treated with immunotherapeutic agents demonstrate response patterns that are not described in the conventional criteria [18]. A specific immunotherapy update, based on the WHO criteria, the immune-related Response Criteria (irRC), was therefore developed, and published in 2009 (Table 2) [14]. These criteria still require validation via correlation with clinical treatment outcomes.

### 2.2. Functional Imaging with MRI

MRI uses strong magnetic fields and radiofrequency pulses to create an image with excellent spatial resolution and tissue contrast [5]. Following two weeks of treatment, MRI was able to differentiate patients with mCRC who were sensitive to treatment with cetuximab and panitumumab from those who were resistant [22]. This early response, defined as tumour shrinkage ≥10% at week 2, detected by MRI represented a reliable early indicator of clinical outcome in terms of progression-free survival, overall survival, and disease control rate [22].

In contrast to CT, which is predominantly a structural, morphological and anatomical technique, MRI is a versatile technique that reveals functional data in addition to structural and anatomical information. In particular, dynamic contrast-enhanced (DCE)-MRI and diffusion-weighted (DW)-MRI may be used to evaluate biological and functional effects of treatment [19]. Assessment of tumour function offers the opportunity to study tumour pathophysiology, heterogeneity and may also predict clinical outcomes, particularly in the setting of novel adjuvant therapies [23].

**Figure 1:**
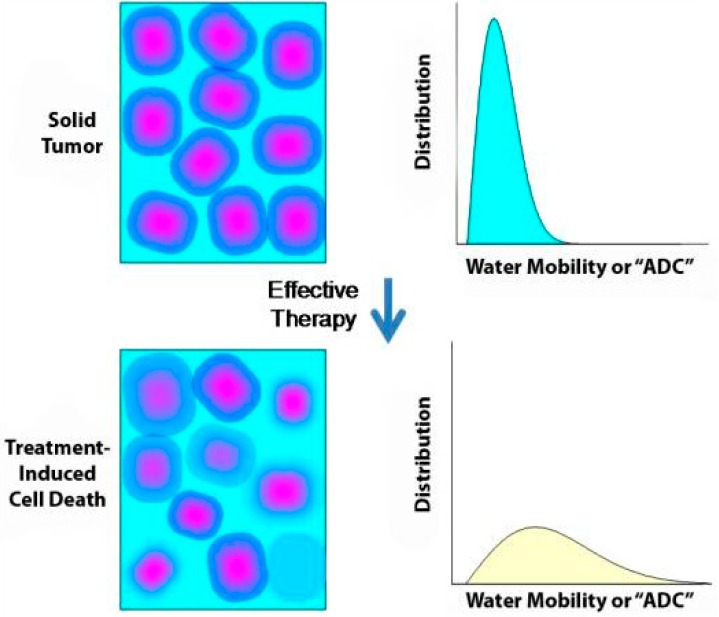

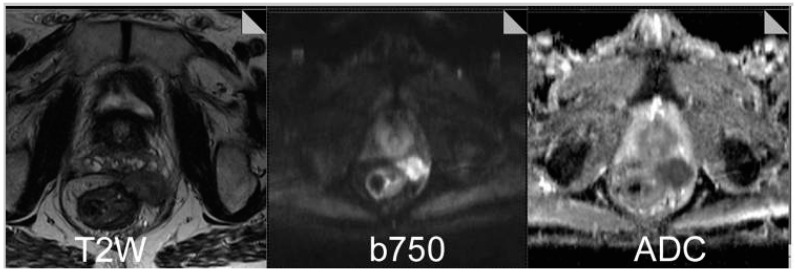
Restricted diffusion within rectal cancer with extension into the perirectal space. T2-weighted image demonstrate a well-circumscribed lesion in the perirectal space. Diffusion-weighted image obtained at a b value of 750 demonstrates a high signal, and corresponding ADC map demonstrates relatively restricted diffusion within the tumour. Figure reproduced with permission from Padhani et al. [24].

DW-MRI derives its contrast from differences in the random movement (“diffusion”) of water protons within a given tissue, which is mainly dependent on cellular density. In tissues with low cellularity, water protons can move relatively freely in the extracellular tissue space, resulting in a low DW-MRI signal. In contrast, tissues with a high cellularity, e.g., a tumour, have a smaller extracellular space, resulting in restricted proton diffusion and a high DW-MRI signal. The degree of proton diffusion can be quantified via the “Apparent Diffusion Coefficient” (ADC), which indirectly reflects the cellular tissue structure (cellularity, Figure 1).

DW-MRI is increasingly being used in oncology as a contrast mechanism [25] with the ability to identify early tumour changes [24] and complete tumour responses in patients with rectal cancer [26]. Because treatment-induced cellular death and vascular changes can both precede changes in lesion size, DW-MRI might be a useful biomarker of treatment outcome for vascular disruptive drugs and therapies that induce apoptosis [24]. The predictive value of DW-MRI has been demonstrated by liver metastases with a high ADC at baseline responding poorly to chemotherapy. Such metastases are commonly associated with the necrosis and loss of cell membrane integrity that suggest an aggressive phenotype [27].

Therapy-induced changes in tumour size are often preceded by changes in perfusion parameters, such as permeability, blood volume, and blood flow [15]. Since capillary perfusion determines the delivery of drugs to tumour cells, recent studies have used the in vivo measurement of capillary perfusion by DCE-MRI as a surrogate marker for measuring the efficacy of bevacizumab-containing chemotherapy regimens [15]. This makes DCE-MRI an attractive modality for evaluating anti-angiogenic cancer therapies, as the rapid acquisition of images before and after intravenous contrast media administration can be used to assess changes in tumour vasculature [20] and predict tumour shrinkage [23]. DCE-MRI can also monitor perfusion changes in response to anti-angiogenic agents in conjunction with CT [15] and ultrasound [28]. However, the question remains whether the observed perfusion changes are predictive for treatment response, or whether they reflect a prognostic tumour phenotype.

### 2.3. Metabolic Imaging with FDG-PET/CT

The glucose analogue ^18^F-fluorodeoxyglucose (FDG) is differentially taken up by malignant cells due to their higher glucose metabolism [19]. This phenomenon may be used to detect both short-term and long-term tumour responses, which are either not apparent with CT or precede a significant decrease in tumour size, by weeks or months [29]. Conversely, a lack of a metabolic response can indicate primary resistance to therapy, while re-emergence of metabolic activity within a tumour site following a period of therapeutic response indicates secondary resistance [29].

When assessed by FDG-PET/CT, metabolic response to chemotherapy correlates well with clinical response, tumour biology and disease-free survival in mCRC [30]. Changes in FDG uptake can be detected after a single course of chemotherapy, with the ability to discriminate mCRC tumours unlikely to respond to treatment [31]. FDG-PET responses have been observed in gastrointestinal stromal tumours as early as 24 h after a single dose of imatinib [29]. The sensitivity of tumour detection by FDG-PET/CT depends on the avidity of the tumour cells for FDG, which is strongly linked to tumour grade (aggressiveness) and cellularity. Metastatic CRC is generally highly avid at baseline, except in cases of mucinous tumours, which may not be detected by a FDG-PET/CT scan. Other digestive tumours that can result in false-negative results are low grade neuroendocrine tumours, well differentiated hepatocellular carcinoma, non-mass forming gastric tumours, and mucinous or cystic pancreatic tumours.

**Figure 2:**
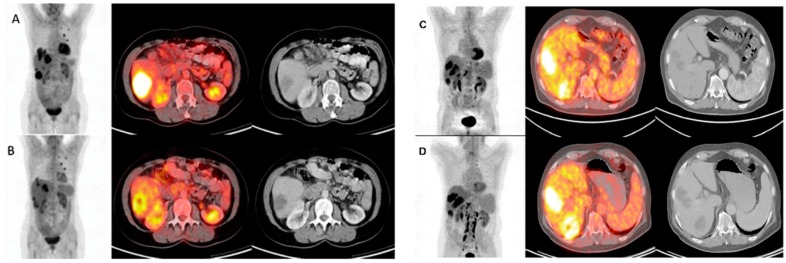
FDG-PET/CT images before (**A** and **C**) and 4 weeks after (**B** and **D**) ^90^Y-microsphere radioembolisation in liver-dominant mCRC; (**A** and **B**) The illustrated metabolic response was associated with a survival of 12 months after treatment; (**C** and **D**) This metabolic non-responder survived 5 months after treatment. Figure reproduced with permission from Sabet et al. [35].

Integrated FDG-PET/CT combines metabolic and anatomical imaging to improve sensitivity and specificity of tumour detection [10], making it an ideal tool to evaluate responses to antineoplastic therapies (Figure 2) [19]. A number of studies have demonstrated improved diagnostic accuracy of integrated FDG-PET/CT compared with stand-alone FDG-PET in a variety of tumour types, including CRC [10]. FDG-PET/CT has proven to be valuable for evaluating responses to chemotherapy and especially for targeted treatments [32]. For example, in a multicentre setting using standardised FDG-PET/CT procedures, early metabolic response was assessed following a combination of sorafenib and capecitabine in chemorefractory mCRC patients as part of the SoMore trial [33,34]. After one single treatment cycle (week 3), FDG-PET/CT could differentiate responsive from unresponsive lesions [33]. Findings such as this may support treatment decisions in the future, although further investigation is required.

The addition of FDG-PET to CT provides complementary metabolic information that enables the detection of malignant disease at unexpected sites or in morphologically normal structures that may be easily overlooked on cross-sectional imaging [10]. FDG-PET/CT has therefore become an established imaging modality recommended for preoperative staging and the detection of mCRC recurrence [32]. As with all novel modalities, reproducibility is required for comparison of response rates between trials [32]. Unlike CT scans, there is no validated, definitive classification scheme for evaluating tumour response with FDG-PET/CT [7]. A fundamental standardisation and consensus on response quantification of FDG-PET/CT methodology is therefore needed. A recent comparison of criteria developed by the European Organization for Research and Treatment of Cancer (EORTC) and PET Response Criteria in Solid Tumours (PERCIST) found the latter to be less ambiguous due to clear definitions and therefore deemed easier to use [32]. One of the major issues is the presence of interlesional tumour response heterogeneity, which is observed on FDG-PET/CT scans of more than 50% of patients with metastatic disease and has a negative predictive and prognostic impact [31,33]. The existing response criteria (both anatomical and metabolic, including those from WHO, EORTC, RECIST and PERCIST) perform an averaging or summing of a selection of target lesions, so most relevant information about the presence of individual treatment resistant lesions is lost. Therefore, new FDG-PET response criteria have been proposed that focus on the presence and proportion of treatment resistance (identification of metabolically stable or progressing lesions) than on treatment responsiveness [31,32,33,34].

An alternative approach to modifying response criteria by imaging modality is modifying according to tumour type. The evolution of imaging modalities has driven the development of response criteria specifically for glioblastoma [36] and renal cell carcinoma [37], and the modification of RECIST for hepatocellular carcinoma [38].

In addition to FDG, various PET radiotracers are establishing roles in oncology, particularly to image therapeutically-targeted intracellular molecular processes that characterise malignancy [10]. Potential indicators of cellular proliferation include ^18^F-FLT, ^11^C-choline and ^18^F-choline. Tracers such as ^15^O-water and ^18^F-FMISO indicate perfusion and hypoxia, respectively; hypoxia is known to contribute to chemotherapy and radiotherapy resistance, leading to angiogenesis and increased metastatic potential [10].

Intensity modulated radiotherapy allows a radiation field to be varied dynamically during treatment, so different structures receive different doses, i.e., a simultaneous integrated boost can treat the primary tumour with a higher radiation dose than the draining lymph nodes, whilst minimizing dose to surrounding normal tissues. A relevant imaging biomarker for radio-resistance, as described above, could be used to determine uptake and identify any differentials within the tumour, thus enabling appropriate dosing of the primary. Also, accurate daily MRI-based adaptive imaging may enable sophisticated dose-painting within small areas of the tumour meaning that resistant areas can be selectively dose-escalated.

A novel approach of molecular imaging using PET/CT is the use of radiolabelled antibodies or antibody fragments. For example, the development of ^89^Zr-cetuximab allows valuable in vivo assessment of epidermal growth factor receptor bio-distribution and bio-availability. This could provide the opportunity to select patients likely to benefit from treatment, identify dose-limiting tissues and optimise therapeutic treatment planning [39]. A recent study of ten patients with wild-type *RAS* mCRC showed a strong correlation between ^89^Zr-cetuximab tumour uptake and clinical response: four-out-of-six patients with ^89^Zr-cetuximab uptake experienced meaningful clinical benefit, while progressive disease was observed in three-out-of-four patients without ^89^Zr-cetuximab uptake [40]. Trials are underway to further explore the relationship between ^89^Zr-cetuximab uptake and treatment response (NCT01691391) [41] as a guide to dose escalation and, ultimately, to create a selection tool to predict cetuximab response (the IMPACT-CRC trial, NCT02117466) [42].

## 3. Rectal Cancer

Recent advances in therapy guidance by imaging and pathology have significantly changed treatment strategies for rectal cancer [7], which comprise approximately a third of all CRC cases [43]. Preoperative assessment of the depth of cancer invasion into and beyond the rectal wall, involvement of the mesorectal fascia, pelvic organs and mesorectal and lateral pelvic nodes is crucial to determine appropriate treatment [44].

Multiple modalities are available for the staging of rectal cancer, including digital examination, endorectal ultrasonography (ERUS), CT and MRI [44]. The most established and preferred modality for the initial staging of rectal cancer is MRI, which can accurately assess tumour size, the extent of tumour protrusion into the surrounding structures, and the distance from the extramural part of the tumour to anatomic landmarks such as the mesorectal fascia [45].

In one meta-analysis of preoperative staging modalities in rectal cancer, the overall T-stage accuracy of MRI was 82%, sensitivity 86% and specificity 77%. For N staging, the accuracy was 82%, sensitivity 82% and specificity 83% [46]. These values have been improved further, with one study including morphological criteria of nodes to achieve an N-stage sensitivity of 85% and a specificity of 98% [47]. MRI has been found to be superior to ERUS and digital rectal examination in terms of clinical benefit, cost-effectiveness, assessment of invasion depth, lymph node involvement, and circumferential resection margin status. This is, in part, due to the excellent resolution of MRI, which can depict the entire mesorectum in a superior manner to CT [5]. ERUS remains the modality of first choice for staging superficial T1 tumours [48].

The accuracy of CT in evaluating rectal cancer is limited by its inability to distinguish layers of the bowel wall [46]. CT is especially limited for local staging of low rectal tumours due to its inherent low soft-tissue contrast, which does not allow for accurate approximation of the extramural extent of the tumour (unless there is gross invasion of adjacent organs; T4) and even in these cases many false-positive cases are reported [5].

Recurrent disease is more common in the rectum than the colon and can result in pain, immobility, and prolonged hospitalisation. Detection of recurrent disease relies on the challenging distinction between tumour and scar tissue related to recent surgery or radiation. In patients who show symptomatic disease and/or rising carcinoembryonic antigen (CEA) during surveillance after rectal cancer surgery, equivocal CT findings may be aided by FDG-PET/CT imaging to improve the detection of a recurrence [5].

## 4. Hepatic Metastatic Disease

The prognosis of patients with CRC is amongst others dependent upon the presence or absence of metastatic spread to the liver [49]. Early detection of liver metastases is also of fundamental importance for achieving disease control [50], particularly as disease with limited spread can be resected for cure [3]. The goals of imaging in this context are to identify the location of all metastatic tumours, determine the feasibility of local resection, exclude the presence of extrahepatic tumour sites, and evaluate the possibility of adjuvant therapy [49]. Ultrasound, CT, MRI and FDG-PET/CT are all used to identify hepatic metastases [49].

Historically, the sensitivity of ultrasound to detect metastases was low and variable (50%–76%) due, in part, to limited contrast between liver lesions and the liver parenchyma [50]. However, the development of ultrasound contrast agents has dramatically improved the detection of liver metastases, with improved specificity (compared to baseline ultrasound) and sensitivity (comparable to CT) [50]. When considered alongside the low price per examination and a favourable safety profile, these factors present contrast-enhanced ultrasound as a significant advancement, particularly for the detection and characterisation of focal liver lesions [51]. One potential disadvantage of contrast-enhanced ultrasound is operator-dependent reliability and reproducibility [52]. Contrast enhanced ultrasound also currently lacks widespread availability, with use limited to expert centres at least in some countries.

**Figure 3:**
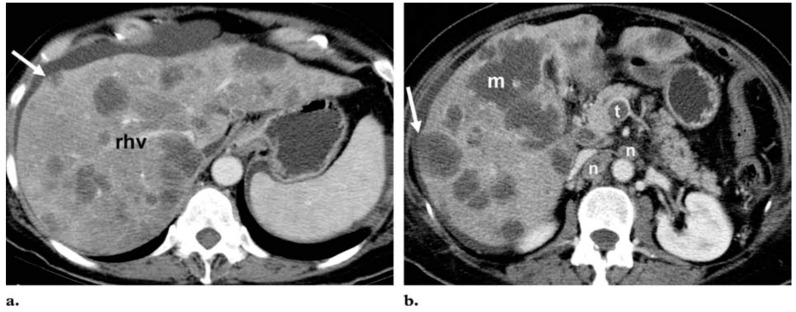
Differentiation of metastases from fat deposition in the liver. Axial portal venous phase contrast-enhanced CT images at the level of the right hepatic vein *(rhv)* (**a**) and the pancreatic head (**b**) show innumerable hypoattenuated lesions throughout the liver. Most of the lesions are round or oval, but the largest (*m* in **b**) has a geographic configuration. Because of their low attenuation (<40 HU), the lesions might be mistaken for multifocal fat deposition; however, the mass effect of the lesions, which produces bulging of the liver surface (arrow) and compression of the right hepatic vein, as well as the multiplicity of lesions, their predominant round or oval shape, the thrombus (*t* in **b**) in the superior mesenteric vein, and numerous heterogeneous lymph nodes (*n* in **b**), are suggestive of malignancy. The lesions were identified as hematogenous metastases from pancreatic adenocarcinoma. Figure reproduced with permission from Hamer et al. [55].

CT is still the preferred method to stage and evaluate the response of CRC with liver metastases both in routine clinical practice and in clinical trials [19]. A survey of 67 Dutch hospitals found CT to be the preferred modality for staging liver disease in patients with CRC [53]. In contrast, MRI was only used for the evaluation of the liver disease in 12 of 20 hospitals (60%) [53]. The various forms of CT scanning have gained extensive use due to reproducibility and widespread distribution [19]. Multidetector CT offers the advantage of volumetric acquisition (using a voxel-based approach), which helps generate high-quality reformatted images in multiple planes for improved detection of small lesions and accurate segmental localisation of lesions [15]. In addition to helping determine progression-free survival, CT is useful for monitoring the effects of adjuvant therapy on metastatic disease due to high sensitivity in determining the size of lesions and excellent determination of visceral involvement [7]. However, the detection of liver metastases using CT can be difficult in the presence of fatty liver (Figure 3), when MRI is more appropriate [54].

Increased soft-tissue resolution makes MRI an invaluable tool in the assessment of liver lesions [15]. Several advances have had a major impact on imaging of the liver, including progress in MRI hardware and software, and contrast enhancement, which is routinely used to evaluate colorectal metastases to the liver [5]. MRI is considered superior to CT and FDG-PET/CT for the detection and characterisation of small liver lesions and for liver evaluation, particularly since the development of DW-MRI [30].

FDG-PET/CT can effectively detect extrahepatic disease and has higher sensitivity (64% vs. 89%) and specificity (70% vs. 90%) than CT in this setting [30,56]. FDG-PET/CT has therefore been recommended for use in a metastatic workup for CRC in the setting of an elevated CEA, when there is concern for distant metastases [7]. Performing FDG-PET/CT in addition to conventional imaging can further support decision-making: one study of 150 patients with mCRC found the addition of FDG-PET to CT resulted in the avoidance of an unnecessary laparotomy in a significant proportion of patients (38%) [57]. The expansion of FDG-PET/CT to evaluate the response of metastatic disease is also a growing area of research [31,32,33].

## 5. Limitations and Challenges of Assessing Treatment Response

The limitations of imaging modalities in CRC include technical, economical and logistical challenges, along with a lack of robust evidence for standardisation and formal guidance. In-house dedicated expertise is a prerequisite for guaranteeing safe and adequate use of imaging in patient management. Careful integration of clinical data with diagnostic information from different imaging modalities is required in a multidisciplinary meeting involving expert clinicians, radiologists and nuclear medicine physicians.

Although highly sensitive, the specificity of CT is region-specific and can be relatively poor [5], for example when staging rectal cancer [7]. CT measurements are also operator-dependent and discrepancies have been reported even among experienced physicians (15%–40% difference in response detection) [7]. Although CT historically lacked the ability to characterise tumour heterogeneity and tumour evolution over time, [7] voxel-based approaches may address spatial heterogeneity within the tumour mass. Such techniques are also used in conjunction with other modalities such as DW-MRI [9]. By comparing pre- and post-treatment ADC maps, the use of voxels allows quantification of diffusion changes that can be superimposed on MRI anatomical maps to reveal spatial information within the context of a 3-dimensional view of the tumour [9].

MRI is primarily being used in a surveillance role or for differentiating benign from malignant disease, with a higher sensitivity and specificity than CT for the detection of colorectal liver metastases [28]. However, the higher cost of MRI and limited ability to detect lung metastases preclude its use over CT for routine surveillance. It is therefore reserved for equivocal findings, as a problem solver before liver surgery or thermal ablation are considered [28].

Higher costs and limited availability of FDG-PET/CT compared with contrast-enhanced CT may have been responsible for an initial lag in uptake for evaluating CRC [58]. However, this delay has been followed by an exponential increase in understanding, use, and reimbursement for PET [5], which has now been superseded by fusion technologies such as FDG-PET/CT [59]. Yet, according to the latest guidelines from the French Digestive Oncology Federation, the National Comprehensive Cancer Network, and the European Society for Medical Oncology, FDG-PET/CT is not routinely indicated for initial staging, therapeutic assessment or follow-up in CRC [30]. This may reflect the limited specificity of FDG-PET. False-positive FDG uptake is well recognised: 5%–8% of CRC patients are reported to be falsely upstaged during preoperative staging by FDG-PET/CT scans to detect disease recurrence [59]. This may be due to inflammation or surgery [15], which may also be responsible for false-positives from other modalities such as DW-MRI.

False-negative results may also occur with FDG-PET/CT, which can be caused by hypometabolic (low grade activity), infracentrimetric or necrotic lesions [15,25]. However, FDG-PET/CT can be performed in addition to MRI in the case of resectable metastatic disease (for initial workup or recurrence) to exclude the presence of extrahepatic metastatic sites [30].

The current functional methods for response assessment suggested by RECIST v1.1 include FDG-PET/CT and DCE-MRI [13]. FDG-PET/CT is relatively expensive and the differentiation provided between residual tumour and inflammation is often difficult using tracers such as ^18^F-FDG. DCE-MRI provides information on changes in vascularisation of the tumour during treatment but image analysis is relatively complicated and therefore not suited for daily clinical practice [9].

Other fusion technologies are also being developed: hybrid PET-MRI machines have recently become commercially available allowing true functional imaging with simultaneously-acquired dynamic PET and MRI data [59]. This may address several of the limitations of FDG-PET/CT by allowing better soft tissue evaluation, more detailed T-staging, improved characterisation of small hepatic lesions, and providing greater anatomical detail for surgical planning while minimising radiation exposure [59].

## 6. Summary and Conclusions

Significant advances have been made in imaging technologies over the last twenty years. Roles have been established in the screening, diagnosis, staging, management and follow-up of CRC. Each modality comes with its inherent advantages and limitations, which we must understand to utilise the technology most appropriately. Examples include the detection of precancerous minute adenomas using the sensitivity of colonoscopy, accurate staging and restaging of rectal cancer using the resolution of MRI, and the detection of recurrent disease using CT and PET in equivocal cases.

In this era of personalised cancer care, imaging plays a critical role in the management of patients with mCRC. As newer agents provide therapeutic benefit by mechanisms other than size reduction the evolution of response criteria is of utmost importance. The availability of new drug classes also places increasing importance on the early identification of treatment response in order to prevent side effects, reduce the costs of futile treatment, and prevent delays in beginning a second-line, potentially effective, therapy.

Our understanding of the development and progression of CRC has improved dramatically, yet challenges still exist for the application of imaging technologies in staging, stratification and monitoring. Advances in imaging technology need to be clinically realised in a routine and robust manner. When considered in combination with the progress in treatment and infrastructure, imaging technology will play an essential role in reducing the mortality of this preventable and manageable cancer.

## Figures and Tables

**Table 1:** RECIST response criteria, version 1.1. [13].

Grade	Response Criteria
Complete response	Disappearance of all target lesions. Any pathological lymph nodes (whether target or non-target) must have reduction in short axis to <10 mm.
Partial response	At least a 30% decrease in the sum of diameters of target lesions, taking as reference the baseline sum diameters.
Progressive disease	At least a 20% increase in the sum of diameters of target lesions, the appearance of one or more new lesions is also considered progression
Stable disease	Neither sufficient shrinkage to qualify for partial response nor sufficient increase to qualify for progressive disease

**Table 2:** Comparison between WHO criteria and the irRC [14].

	WHO	irRC *
New, measurable lesions (i.e., ≥5 × 5 mm)	Always represent PD	Incorporated into tumor burden
New, nonmeasurable lesions (i.e., < 5 × 5 mm)	Always represent PD	Do not define progression (but preclude irCR)
Non-index lesions	Changes contribute to defining BOR of CR, PR, SD, and PD	Contribute to defining irCR (complete disappearance required)
Complete response	Disappearance of all lesions in two consecutive observations not less than 4 wk apart	Disappearance of all lesions in two consecutive observations not less than 4 weeks apart
Partial response	≥50% decrease in SPD of all index lesions compared with baseline in two observations at least 4 weeks apart, in absence of new lesions or unequivocal progression of non-index lesions	≥50% decrease in tumor burden compared with baseline in two observations at least 4 weeks apart
Stable disease	50% decrease in SPD compared with baseline cannot be established nor 25% increase compared with nadir, in absence of new lesions or unequivocal progression of non-index lesions	50% decrease in tumour burden compared with baseline cannot be established nor 25% increase compared with nadir
Progressive disease	At least 25% increase in SPD compared with nadir and/or unequivocal progression of non-index lesions and/or appearance of new lesions (at any single time point)	At least 25% increase in tumour burden compared with nadir (at any single time point) in two consecutive observations at least 4 weeks apart

* These criteria still require validation via correlation with clinical treatment outcomes.
